# Predicting risk of the subsequent early pregnancy loss in women with recurrent pregnancy loss based on preconception data

**DOI:** 10.1186/s12905-024-03206-9

**Published:** 2024-07-02

**Authors:** Xin Yang, Ruifang Wang, Wei Zhang, Yanting Yang, Fang Wang

**Affiliations:** 1 Department of Reproductive Medicine, Lanzhou University Second Hospital, Lanzhou, 730030 China; 2 Department of Obstetrics and Gynecology, The First Affiliated Hospital, College of Clinical Medicine of Henan University of Science and Technology, Luoyang, 471003 China

**Keywords:** Recurrent pregnancy loss, Prediction model, Pregnancy loss risk

## Abstract

**Background:**

For women who have experienced recurrent pregnancy loss (RPL), it is crucial not only to treat them but also to evaluate the risk of recurrence. The study aimed to develop a risk predictive model to predict the subsequent early pregnancy loss (EPL) in women with RPL based on preconception data.

**Methods:**

A prospective, dynamic population cohort study was carried out at the Second Hospital of Lanzhou University. From September 2019 to December 2022, a total of 1050 non-pregnant women with RPL were participated. By December 2023, 605 women had subsequent pregnancy outcomes and were randomly divided into training and validation group by 3:1 ratio. In the training group, univariable screening was performed on RPL patients with subsequent EPL outcome. The least absolute shrinkage and selection operator (LASSO) regression and multivariate logistic regression were utilized to select variables, respectively. Subsequent EPL prediction model was constructed using generalize linear model (GLM), gradient boosting machine (GBM), random forest (RF), and deep learning (DP). The variables selected by LASSO regression and multivariate logistic regression were then established and compared using the best prediction model. The AUC, calibration curve, and decision curve (DCA) were performed to assess the prediction performances of the best model. The best model was validated using the validation group. Finally, a nomogram was established based on the best predictive features.

**Results:**

In the training group, the GBM model achieved the best performance with the highest AUC (0.805). The AUC between the variables screened by the LASSO regression (16-variables) and logistic regression (9-variables) models showed no significant difference (AUC: 0.805 vs. 0.777, *P* = 0.1498). Meanwhile, the 9-variable model displayed a well discrimination performance in the validation group, with an AUC value of 0.781 (95%CI 0.702, 0.843). The DCA showed the model performed well and was feasible for making beneficial clinical decisions. Calibration curves revealed the goodness of fit between the predicted values by the model and the actual values, the Hosmer–Lemeshow test was 7.427, and *P* = 0.505.

**Conclusions:**

Predicting subsequent EPL in RPL patients using the GBM model has important clinical implications. Future prospective studies are needed to verify the clinical applicability.

**Trial registration:**

This study was registered in the Chinese Clinical Trial Registry with the registration number of ChiCTR2000039414 (27/10/2020).

**Supplementary Information:**

The online version contains supplementary material available at 10.1186/s12905-024-03206-9.

## Background

Pregnancy loss (PL) is defined as the spontaneous demise of a pregnancy before the fetus reaches viability (from the time of conception until 24 weeks of gestation), also referred to as a miscarriage or spontaneous abortion, is one of the common health problems in childbearing women, which impacts 10–15% of clinically recognized pregnancies [1]. And early PL (EPL) is the loss pregnancy before 10 weeks of gestational age, which accounts for more than 80% of all PLs [2, 3]. 1-3% of women of childbearing age will have two or more PLs, known as recurrent pregnancy loss (RPL). Studies have identified various risk factors being related to PL, including age, BMI, ethnicity, previous miscarriages, uterine anatomy abnormalities, chromosomal abnormalities, infection, immune dysfunction, endocrine disturbance, and unhealthy lifestyle [4, 5], but there still approximately 60% of RPL cases remains unexplained, and these cases are referred to as unexplained RPL (URPL) [3].

RPL greatly affects the physical and mental health of couples of childbearing ages [6]. Women with a history of RPL showed more psychological problems during their subsequent pregnancy. Notably, couples must deal with the cumulative emotional effects by the subsequent RPL [7]. Whereas, some patients come to the hospital seek for help when they had a pregnancy-related concern (bleeding, abdominal pain, or worsening anxiety due to prior miscarriages or ectopic pregnancy), at which point the risk of pregnancy loss is increased. In addition, clinicians use clinical and demographic information to predict pregnancy outcomes after a patient becomes pregnant, but some early pregnancy loss is inevitable. For women who have experienced RPL, it is not only important to diagnose and treat them, but also to evaluation of the risk for recurrence which can reduce the subsequent PL rates.

The risk of RPL increased with the number of times of pregnancy loss, and the incidence of pregnancy loss was only 11.6% in women without a history of pregnancy loss. In women with a history of one, two, and three or more pregnancy losses, the probability of subsequent pregnancy loss was 19.8%, 27.7%, and 41.9%, respectively [8]. A population-based study has found that the lowest risk of PL (9.8%) in women aged 25–29 years and the risk of PL increases in women aged 30–35 years, then rises steeply to 33.2% in women aged 40–44 year [8]. The number of previous PLs was another independent risk factor for RPL [9]. Genetic factors may also be involved in the risk of miscarriage. One large genome-wide association study identified four distinct susceptibility loci for sporadic and RPL that have a role in progesterone production, placentation, and gonadotropin regulation [10]. There is research found that women experiencing bleeding without nausea between 6- and 8-weeks’ gestation had an increased risk of clinical pregnancy loss, but bleeding and nausea were not predictive risk factors of clinical pregnancy loss prior to 6 weeks’ gestation [11]. A meta-analysis found that early pregnancy ultrasound markers, including fetal bradycardia, crown rump length, intra uterine hematoma, and mean gestational sac diameter minus could predicting miscarriage in women with diagnosed viable intrauterine pregnancy [12]. In recent years, some novel markers of immune tolerance and angiogenesis in maternal blood have been reported as potential RPL predictors, including immune tolerance proteins galectin-9 (Gal-9) and interleukin (IL)-4, and angiogenesis proteins (vascular endothelial growth factors (VEGF) A, C, and D) [13].

Among these risk factors, some of them are limited by their late appearance or poor temporary availability and it is difficult to comprehensive assessment of the risk of subsequent RPL. Furthermore, most RPL risk assessment use traditional regression models (e.g., logistic regression), which make an implicit assumption that each risk factor is linearly related to PL [14], this may ignore the complex relationships of many risk factors with non-linear interactions, and the predictive performance is always suboptimal. Therefore, exploring an effective prediction model to predict the subsequent EPL for RPL patients is necessary.

Recently, machine learning (ML) methods, has been reported to demonstrate a powerful self-learning ability with improved prediction accuracy [15, 16], and it has been successfully applied to diagnosing diseases and predicting clinical outcomes, such as for in vitro fertilization treatment [15], RPL [16], postpartum hemorrhage [17] and other pregnancy pathological events [18]. Furthermore, the nomogram as a simple statistical visual tool, which is widely used to predict the occurrence of diseases. In this study, we develop and validate a prediction machine learning model based on the preconception demographic information, reproductive history, and clinical blood parameters of admission to identify the risk of subsequent EPL for RPL patients.

## Materials and methods

### Participants

The study population was drawn from a prospective, dynamic cohort, which was carried out at the Department of Reproductive Medicine, Second Hospital of Lanzhou University [19]. The cohort began in September 2019 and enrolled 1050 nonpregnant RPL patients through December 2022. The inclusion criteria were: (1) Have experienced at least two history of PL that meets the diagnostic criteria of the ESHRE; (2) aged 18–42 years. The follow-up period ended in December 2023 and the exclusion criteria were: (1) Patients who were lost to follow-up and who were not yet pregnant; (2) Subsequent pregnancy outcomes are ectopic pregnancy, hydatidiform mole, dysplasia, and current pregnancy < 10 weeks; (3) Subsequent pregnancies were assisted reproductive technology and twin pregnancies. This study was approved by the Ethics Committee of Lanzhou University Second Hospital (Ethical Approval Number: 2019 A-231). All patients provided written informed consent.

### Predictive variables

Demographic information (including age, height, weight, education, ethnic, menarche, menstrual cycle, and pelvic surgery), reproductive history (including total pregnancy numbers, pregnancy loss numbers, induced abortion, live birth, and pregnancy type) were obtained from outpatient medical records and body mass index (BMI) was calculated as weight in kilograms divided by the square of body height in meter. Preconception treatments and subsequent pregnancy outcome was obtained from the follow-up. Each patient was followed up through electronic medical record system and telephone every 6 months after the first visit to track the patient’s pregnancy status, most recently in December 2023. Blood samples obtained at the initial visit in a nonpregnant state in in the morning, when the patient was underwent overnight fasting, and was tested according to the standard manufacturer’s protocols within an hour at our hospital and the blood parameters including 50 indicators. The demographic information and blood test indicators are presented in Table 1.

**Table 1:** The baseline demographic information and blood test indicators of RPL patients between the training and validation group

Variables	All *N* = 605	Training group *n* = 454	Validation group *n* = 151	*P*-value
Subsequent pregnancy				0.834
Ongoing pregnancy (n, %)	465 (76.86%)	348 (76.65%)	117 (77.48%)	
Early pregnancy loss (n, %)	140 (23.14%)	106 (23.35%)	34 (22.52%)	
Age (year)	30.02 ± 3.81	30.07 ± 3.73	29.88 ± 4.05	0.601
BMI (kg/m^2^)	22.12 ± 2.70	22.18 ± 2.73	21.93 ± 2.60	0.324
Total pregnancy numbers	2.78 ± 0.97	2.81 ± 1.02	2.68 ± 0.80	0.126
Pregnancy loss numbers	2.38 ± 0.64	2.40 ± 0.66	2.30 ± 0.58	0.120
PLs stratification				0.115
2	423 (69.92%)	312 (68.72%)	111 (73.51%)	
3	145 (23.97%)	109 (24.01%)	36 (23.84%)	
≥ 4	37 (6.12%)	33 (7.27%)	4 (2.65%)	
Education				0.336
Primary school (n, %)	20 (3.47%)	14 (3.08%)	6 (3.97%)	
Secondary school (n, %)	167 (27.60%)	124 (27.31%)	43 (28.48%)	
Bachelor degree (n, %)	389 (64.30%)	287 (63.22%)	102 (67.55%)	
Graduate degree (n, %)	28 (4.63%)	25 (5.51%)	3 (1.99%)	
Ethnic				0.601
Han nationality (n, %)	546 (91.00%)	407 (90.65%)	139 (92.05%)	
Others (n, %)	54 (9.00%)	42 (9.35%)	12 (7.95%)	
Menarche (year)	13.49 ± 1.20	13.49 ± 1.23	13.49 ± 1.14	0.871
Menstrual cycle				0.571
Regular (n, %)	527 (87.11%)	394 (86.78%)	133 (88.08%)	
Irregular (n, %)	78 (12.89%)	60 (13.22%)	18 (11.92%)	
Pelvic surgery				0.166
No (n, %)	494 (81.65%)	365 (80.40%)	129 (85.43%)	
Yes (n, %)	111 (18.35%)	89 (19.60%)	22 (14.57%)	
Preconception treatments				0.489
No (n, %)	190 (31.40%)	146 (32.16%)	44 (29.14%)	
Yes (n, %)	415 (68.60%)	308 (67.84%)	107 (70.86%)	
Induced abortion				0.353
No (n, %)	532 (87.93%)	396 (87.22%)	136 (90.07%)	
Yes (n, %)	73 (12.07%)	58 (12.78%)	15 (9.93%)	
Live birth				0.372
No (n, %)	484 (80.00%)	367 (80.84%)	117 (77.48%)	
Yes (n, %)	121 (20.00%)	87 (19.16%)	34 (22.52%)	
Pregnancy type				0.326
Primary (n, %)	474 (78.35%)	360 (79.30%)	114 (75.50%)	
Secondary (n, %)	131 (21.65%)	94 (20.70%)	37 (24.50%)	
TSH (uIU /mL)	2.59 ± 1.40	2.62 ± 1.36	2.50 ± 1.53	0.377
TG-Ab				0.489
Negative (n, %)	481((85.13%)	355 (84.52%)	126 (86.90%)	
Positive (n, %)	84 (14.87%)	65 (15.48%)	19 (13.10%)	
TPO-Ab				0.723
Negative (n, %)	478 (84.60%)	354 (84.29%)	124 (85.52%)	
Positive (n, %)	87 (15.40%)	66 (15.71%)	21 (14.48%)	
IG-G (g/L)	12.94 ± 2.81	12.85 ± 2.95	13.19 ± 2.37	0.225
IG-A (g/L)	2.18 ± 0.73	2.20 ± 0.53	2.13 ± 1.14	0.299
IG-M(g/L)	2.03 ± 0.74	2.03 ± 0.77	2.03 ± 0.67	0.985
C3 (g/L)	1.14 ± 0.20	1.14 ± 0.20	1.13 ± 0.19	0.538
C4 (g/L)	0.26 (0.21–0.32)	0.30 ± 0.33	0.26 ± 0.08	0.169
ANA				0.495
Negative (n, %)	498 (87.99%)	369 (87.44%)	129 (89.58%)	
Positive (n, %)	68 (12.01%)	53 (12.56%)	15 (10.42%)	
ACA				0.892
Negative (n, %)	512 (89.35%)	382 (89.25%)	130 (89.66%)	
Positive (n, %)	61 (10.65%)	46 (10.75%)	15 (10.34%)	
β2GP1				0.899
Negative (n, %)	527 (91.97%)	394 (92.06%)	133 (91.72%)	
Positive (n, %)	46 (8.03%)	34 (7.94%)	12 (8.28%)	
LA				0.394
Negative (n, %)	528 (92.15%)	392 (91.59%)	136 (93.79%)	
Positive (n, %)	45 (7.85%)	36 (8.41%)	9 (6.21%)	
D-dimer (mg/L)	0.18 (0.12–0.28)	0.18 (0.12–0.26)	0.18 (0.13–0.29)	0.718
HCY (umol/L)	11.71 ± 5.97	11.64 ± 5.78	11.92 ± 6.50	0.619
25(OH)D (ng/ml)	12.01 ± 4.63	11.98 ± 4.72	12.11 ± 4.37	0.777
FBG (mmol/L)	4.99 ± 0.44	4.97 ± 0.43	5.03 ± 0.46	0.167
FINS (mU/L)	10.21 (7.13–12.28)	10.46 (6.95–12.25)	9.25 (7.36–12.41)	0.503
HOMA-IR	2.41 ± 1.58	2.43 ± 1.69	2.33 ± 1.14	0.533
FCP (ng/ml)	1.36 ± 0.73	1.38 ± 0.79	1.28 ± 0.49	0.159
2 h-BG (mmol/L)	5.67 ± 1.22	5.70 ± 1.23	5.58 ± 1.18	0.307
2 h-INS (mU/L)	31.03 (23.67–51.64)	31.06 (24.08–51.18)	30.97 (21.63–53.19)	0.601
2 h-CP (ng/ml)	4.48 ± 1.82	4.54 ± 1.80	4.26 ± 1.91	0.130
CHO (mmol/L)	3.86 ± 0.67	3.86 ± 0.70	3.86 ± 0.59	0.973
TG (mmol/L)	0.94 (0.73–1.21)	0.99 (0.73–1.24)	0.90 (0.75–1.12)	0.130
HDL (mmol/L)	1.41 ± 0.31	1.42 ± 0.32	1.38 ± 0.27	0.489
LDL (mmol/L)	2.45 ± 0.59	2.45 ± 0.61	2.45 ± 0.52	0.326
CHR	2.85 ± 0.71	2.84 ± 0.73	2.88 ± 0.66	0.337
THR	0.63 (0.50–0.98)	0.62 (0.50–1.03)	0.63 (0.53–0.85)	0.370
LHR	1.82 ± 0.59	1.82 ± 0.60	1.85 ± 0.56	0.235
WBC (×10^9^)	5.88 ± 2.35	5.94 ± 2.56	5.68 ± 1.50	0.492
NE# (×10^9^)	3.69 ± 1.31	3.71 ± 1.31	3.64 ± 1.31	0.979
LY# (×10^9^)	1.71 ± 0.47	1.73 ± 0.47	1.65 ± 0.46	0.206
MO# (×10^9^)	0.27 ± 0.09	0.27 ± 0.09	0.26 ± 0.08	0.297
RBC (×10^12^)	4.60 ± 0.39	4.59 ± 0.38	4.65 ± 0.42	0.267
HGB (g/L)	140.95 ± 12.66	140.90 ± 12.73	141.11 ± 12.50	0.962
PLT (×10^9^)	222.59 ± 59.09	224.11 ± 59.01	217.82 ± 59.34	0.175
LWR	0.30 ± 0.08	0.30 ± 0.07	0.31 ± 0.08	0.667
NLR	2.29 ± 1.00	2.27 ± 0.97	2.36 ± 1.10	0.451
NMR	14.24 ± 4.91	14.18 ± 4.90	14.44 ± 4.97	0.201
LMR	6.63 ± 2.08	6.61 ± 2.08	6.71 ± 2.09	0.464
PWR	40.12 ± 12.16	40.14 ± 12.26	40.03 ± 11.88	0.744
PNR	66.38 ± 26.91	66.36 ± 26.89	66.46 ± 27.08	0.762
PLR	138.07 ± 45.69	139.00 ± 47.89	135.20 ± 38.09	0.307
PMR	883.63 ± 321.17	883.04 ± 325.20	885.49 ± 309.59	0.456
ALT (U/L)	17.85 ± 13.54	18.01 ± 14.50	17.32 ± 9.94	0.630
AST (U/L)	22.76 ± 9.31	22.84 ± 9.92	22.52 ± 7.06	0.745
AST/ALT	1.62 ± 0.95	1.64 ± 0.97	1.57 ± 0.91	0.517
SUR (mmol/L)	4.38 ± 1.16	4.37 ± 1.14	4.41 ± 1.22	0.770
SCR (µmol/L)	54.53 ± 7.72	54.74 ± 7.59	53.85 ± 8.15	0.278
SUA (µmol/L)	266.55 ± 60.97	269.38 ± 59.83	257.49 ± 63.91	0.067

Continuous variables are expressed in mean ± standard deviation (SD) or median (25th–75th percentiles). Categorical variables were expressed as frequencies (percentages)

*Abbreviations:* *BMI* Body mass index, *TSH *Thyroid stimulating hormone, *TG-Ab* Thyroglobulin antibody, *TPO-Ab* Thyroid peroxidase antibodies, *ANA* Antinuclear antibody, *ACA* Anti cardiolipin antibody, *β2GP1* β2-glycoprotein 1, *LA* Lupus anticoagulant, *IgG* Immunoglobulin G, *IgA* Immunoglobulin A, *IgM* Immunoglobulin M, *C3* Complement C3, *C4* Complement C4, *HCY* Homocysteine, *25(OH)D* 25-hydroxy-vitamin, *FBG* Fasting blood glucose, *FINS* Fasting insulin, *HOMA-IR* Homeostasis model assessment of insulin resistance, *FCP* Fasting C-peptide, *2 h-BG* 2-hour postprandial blood glucose, *2 h-INS* 2-hour postprandial insulin, *2 h-CP* 2-hour postprandial C-peptide, *CHO* Cholesterol, *TG *Triglyceride, *HDL* High-density lipoprotein, *LDL* Low-density lipoprotein, *CHR* Cholesterol to high-density lipoprotein ratio, *THR* Triglyceride to high-density lipoprotein ratio, *LHR* Low-density lipoprotein to high-density lipoprotein ratio, *WBC* White blood cell, *NE* Neutrophilic, *LY* Lymphocyte, *MO* Monocytes, *RBC* Red blood cell, *HGB* Hemoglobin, *PLT* Platelet, *LWR* Lymphocyte to white blood cell ratio, *NLR* Neutrophilic to lymphocyte ratio, *NMR *Neutrophilic to monocytes ratio, *LMR* Lymphocyte to monocytes ratio, *PWR* Platelet to white blood cell ratio, *PNR* Platelet to neutrophilic ratio, *PLR* Platelet to lymphocyte, *PMR* Platelet to monocytes ratio, *ALT* Alanine Aminotransferase, *AST* Aspartate Transaminase, *AST/ALT* Aspartate Transaminase to alanine Aminotransferase, *SUR* Serum urea, *SCR* Serum creatinine, *SUA* Serum uric acid

### Outcomes

The primary outcomes of this analysis included subsequent EPL and ongoing pregnancy (OP). EPL was defined as pregnancy less than 10 weeks of gestational age, including biochemical pregnancy. OP was defined as pregnancy beyond 10 weeks of gestational age.

### Statistical analyses

Continuous variables are described by mean ± standard deviation or median (interquartile range), categorical variables are described using percentages. Independent sample T test, Mann–Whitney–Wilcoxon test, chi-square test and Fisher’s exact test were used appropriate. Multiple imputations were performed for a few missing variables (details of statistical methods for each variable, the number and percentage of missing data are presented in Supplementary Table 1). We generated five data sets by multiple imputations, and sensitivity analysis showed that these five data sets were not significantly different from the original data, the results are presented in Supplementary Table 2. All analyses were performed using Empower(R) (www.empowerstats.com, X&Y solutions, inc.Boston MA) and R (http://www.R-project.org).

**Table 2:** The baseline demographic information and blood test indicators of RPL patients between subsequent EPL and OP in the training group

Variables	OP *n* = 348	EPL *n* = 106	*P*-value
Age (year)	29.83 ± 3.48	30.86 ± 4.37	0.013*
BMI (kg/m^2^)	22.03 ± 2.77	22.65 ± 2.55	0.043*
Total pregnancy numbers	2.76 ± 0.95	3.00 ± 1.20	0.033*
Pregnancy loss numbers	2.35 ± 0.60	2.56 ± 0.82	0.005*
Education			0.723
Primary school (n, %)	10 (2.87%)	4 (3.77%)	
Secondary school (n, %)	92 (26.44%)	32 (30.19%)	
Bachelor degree (n, %)	221 (63.51%)	66 (62.26%)	
Graduate degree (n, %)	20 (5.75%)	5 (4.72%)	
Ethnic			0.974
Han nationality (n, %)	311 (90.67%)	96 (90.57%)	
Others (n, %)	32 (9.33%)	10 (9.43%)	
Menarche (year)	13.46 ± 1.24	13.59 ± 1.19	0.352
Menstrual cycle			0.346
Regular (n, %)	298 (85.63%)	96 (90.57%)	
Irregular (n, %)	48 (13.79%)	12 (13.21%)	
Pelvic surgery			0.058
No (n, %)	273 (78.45%)	92 (86.79%)	
Yes (n, %)	75 (21.55%)	14 (13.21%)	
Preconception treatments			0.796
No (n, %)	113 (32.47%)	33 (31.13%)	
Yes (n, %)	235 (67.53%)	73 (68.87%)	
Induced abortion			0.002*
No (n, %)	313 (89.94%)	83 (78.30%)	
Yes (n, %)	35 (10.06%)	23 (21.70%)	
Live birth			0.449
No (n, %)	284 (81.61%)	83 (78.30%)	
Yes (n, %)	64 (18.39%)	23 (21.70%)	
Pregnancy type			0.574
Primary (n, %)	278 (79.89%)	82 (77.36%)	
Secondary (n, %)	70 (20.11%)	24 (22.64%)	
TSH (uIU /mL)	2.59 ± 1.36	2.72 ± 1.35	0.379
TG-Ab			0.474
Negative (n, %)	278 (86.88%)	84 (84.00%)	
Positive (n, %)	42 (13.13%)	16 (16.00%)	
TPO-Ab			0.009*
Negative (n, %)	278 (86.88%)	76 (76.00%)	
Positive (n, %)	42 (13.12%)	24 (24.00%)	
IG-G (g/L)	12.77 ± 3.08	13.12 ± 2.45	0.326
IG-A (g/L)	2.21 ± 0.55	2.16 ± 0.46	0.357
IG-M(g/L)	1.98 ± 0.76	2.19 ± 0.79	0.041*
C3 (g/L)	1.14 ± 0.19	1.14 ± 0.20	0.864
C4 (g/L)	0.26 (0.21–0.32)	0.27 (0.22–0.32)	0.023*
ANA			0.092
Negative (n, %)	283 (88.99%)	86 (82.69%)	
Positive (n, %)	35 (11.01%)	18 (17.31%)	
ACA			< 0.001*
Negative (n, %)	303 (93.52%)	79 (75.96%)	
Positive (n, %)	21 (6.48%)	25 (24.04%)	
β2GP1			0.005*
Negative (n, %)	305 (94.14%)	89 (85.58%)	
Positive (n, %)	19 (5.86%)	15 (14.42%)	
LA			< 0.001*
Negative (n, %)	307 (94.75%)	85 (81.73%)	
Positive (n, %)	17 (5.25%)	19 (18.27%)	
D-dimer (mg/L)	0.17 (0.12–0.25)	0.19 (0.12–0.28)	0.147
HCY (umol/L)	10.70 (9.00-11.90)	11.66 (10.40-14.65)	< 0.001*
25(OH)D (ng/ml)	11.97 ± 4.80	12.00 ± 4.45	0.958
FBG (mmol/L)	4.98 ± 0.43	4.95 ± 0.43	0.569
FINS (mU/L)	10.61 (6.92–12.36)	9.88 (7.34–11.46)	0.704
HOMA-IR	2.45 ± 1.78	2.39 ± 1.35	0.743
FCP (ng/ml)	1.37 ± 0.67	1.43 ± 1.10	0.501
2 h-BG (mmol/L)	5.64 ± 1.21	5.92 ± 1.28	0.035*
2 h-INS (mU/L)	30.00 (24.09–47.17)	41.07 (24.24–65.14)	0.014*
2 h-CP (ng/ml)	4.52 ± 1.72	4.61 ± 2.03	0.642
CHO (mmol/L)	3.86 ± 0.65	3.87 ± 0.85	0.886
TG (mmol/L)	1.01 (0.75–1.18)	0.92 (0.69–1.24)	0.684
HDL (mmol/L)	1.45 ± 0.33	1.31 ± 0.25	< 0.001*
LDL (mmol/L)	2.44 ± 0.58	2.47 ± 0.68	0.652
CHR	2.78 ± 0.73	3.03 ± 0.71	0.002*
THR	0.59 (0.50–0.98)	0.70 (0.49–1.03)	0.125
LHR	1.78 ± 0.60	1.95 ± 0.60	0.009*
WBC (×109)	5.98 ± 2.80	5.80 ± 1.51	0.571
NE# (×109)	3.77 ± 1.38	3.53 ± 1.06	0.143
LY# (×109)	1.74 ± 0.49	1.68 ± 0.41	0.236
MO# (×109)	0.28 ± 0.08	0.27 ± 0.09	0.643
RBC (×1012)	4.59 ± 0.37	4.60 ± 0.41	0.767
HGB (g/L)	140.80 ± 13.21	141.25 ± 11.02	0.771
PLT (×109)	222.87 ± 60.97	228.29 ± 51.96	0.447
LWR	0.30 ± 0.07	0.31 ± 0.07	0.266
NLR	2.28 ± 1.00	2.22 ± 0.85	0.577
NMR	14.26 ± 4.91	13.92 ± 4.89	0.567
LMR	6.52 ± 2.01	6.90 ± 2.32	0.133
PWR	39.74 ± 12.19	41.51 ± 12.47	0.232
PNR	64.69 ± 23.17	72.08 ± 36.51	0.024*
PLR	138.31 ± 46.96	141.35 ± 51.16	0.603
PMR	870.28 ± 324.61	926.72 ± 325.29	0.155
ALT (U/L)	12.33 ± 4.67	12.55 ± 4.16	0.694
AST (U/L)	3.06 ± 1.82	3.18 ± 1.64	0.584
AST/ALT	9.32 ± 4.35	9.37 ± 4.06	0.920
SUR (mmol/L)	18.52 ± 15.86	16.36 ± 8.52	0.227
SCR (µmol/L)	22.93 ± 10.87	22.56 ± 5.86	0.768
SUA (µmol/L)	1.63 ± 1.02	1.65 ± 0.77	0.859

Continuous variables are expressed in mean ± standard deviation (SD) or median (25th–75th percentiles). Categorical variables were expressed as frequencies (percentages). ^*^P value < 0.05

*Abbreviations* Consistent with Table 1

### Variable selection

The dataset of the RPL women was randomly split into the development (75%) and validation (25%) groups. Data for 63 variables during pre-pregnancy were obtained from the patient self-reports and electronic medical records. First, in order to create an efficient approach for clinical practice with fewer redundant variables, we performed independent sample t test, Mann–Whitney–Wilcoxon test, chi-square test or Fisher’s exact test at the appropriate time, and then the variables with *P* < 0.05 were used the least absolute shrinkage and selection operator (LASSO) logistic regression algorithm and multivariate regression analysis with the training group to select related features.

### Model training, evaluation, and validation

The features selected by LASSO regression were performed on the training group using the four models including generalize linear model (GLM), gradient boosting machine (GBM), random forest (RF), and deep learning (DP). After the model was established, we used area under the ROC curve (AUC), area under the precision-recall curve (AUCPR), logloss, mean per-class error, root mean square error (RMSE) and mean square error (MSE) to compare the models. And the model with the largest AUC value was selected as the best model. Next, the variables selected by LASSO regression and multivariate logistic regression were established and compared using the best prediction model in the training group. The AUC, calibration curve (Hosmer–Lemeshow test), and clinical decision curve (DCA) were performed to assess the prediction performances and clinical utility of the best model. We further performed an internal validation for the developed prediction model using the validation group. Finally, based on the best predictive features, a nomogram was established to take advantage of fitting a line with a non-linear relationship for the prediction of subsequent EPL.

## Results

### Baseline characteristics

**Fig. 1:**
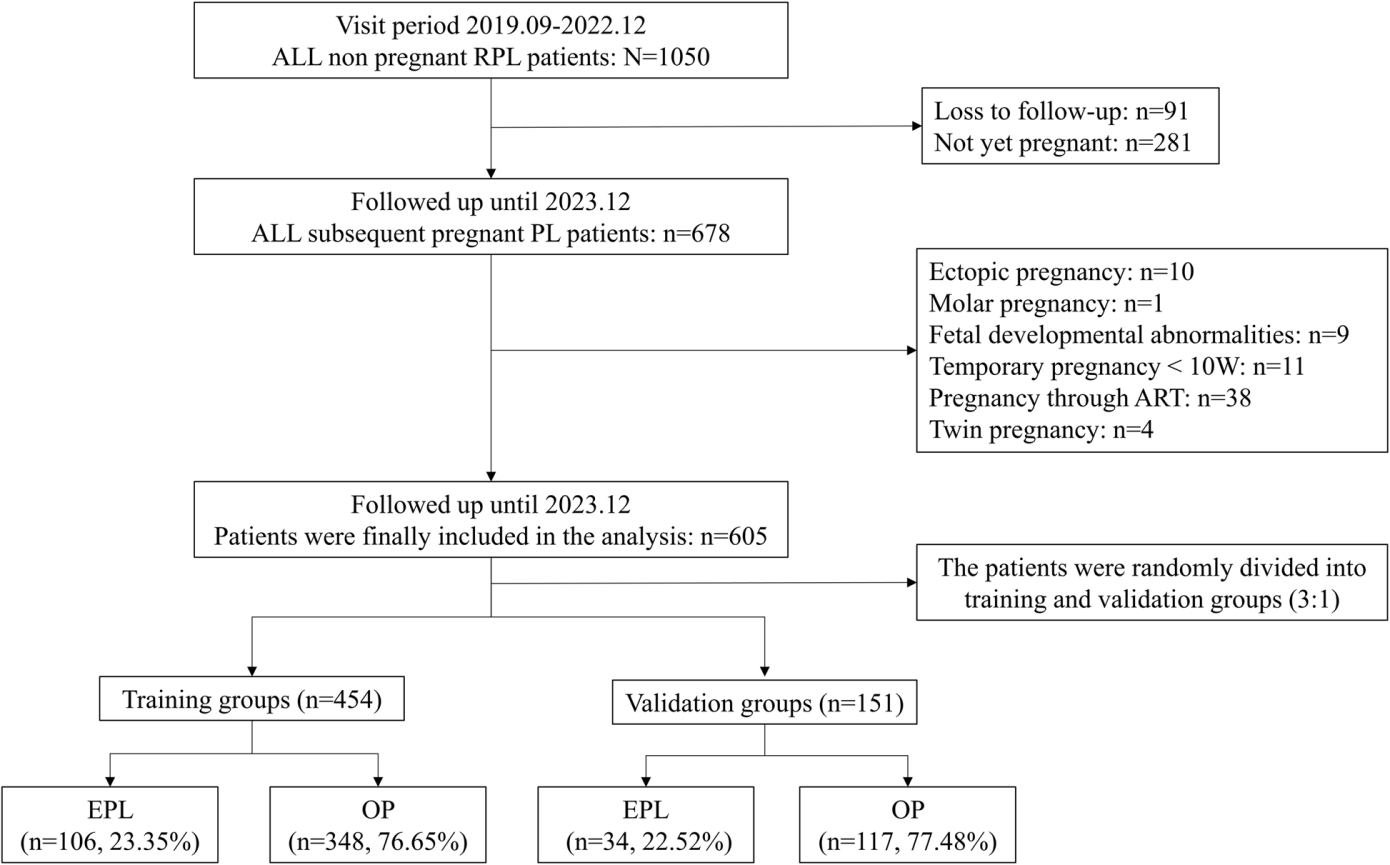
Flow chart for RPL patient selection. RPL: recurrent pregnancy loss; EPL: early pregnancy loss; OP: ongoing pregnancy

Finally, 605 eligible RPL patients were enrolled in this stud, they were randomly divided into training group and validation group by 3:1 ratio, with 454 patients in the training group and 151 patients in the validation group. A flow chart of the process is represented in Fig. 1. Subsequent EPL occurred on 23.14% (140/605) in all patients and 23.35% (106/454) in the training group, 22.52% (34/151) in the validation group. Women with 423 (69.92%), 145 (23.97%), 37 (6.12%) had experienced 2, 3 or ≥ 4 prior pregnancy losses, respectively. No statistically significant difference between training group and validation group (*P* > 0.05) (Table 1).

### Screening of predictors

**Fig. 2:**
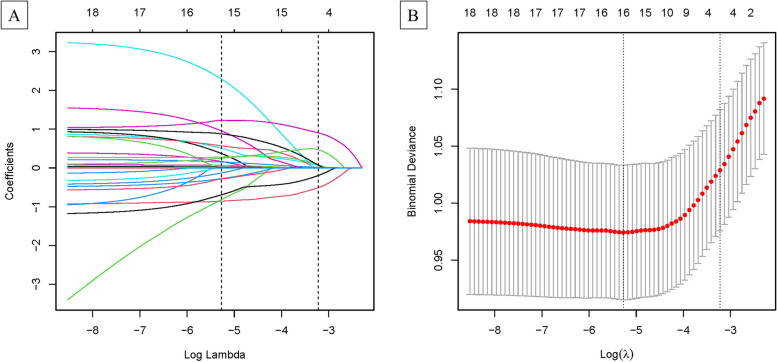
Clinical features selection using the least absolute shrinkage and selection operator (LASSO). **A** Tuning parameter(lambda) selection in the LASSO model used 10-fold cross-validation via minimum criteria. **B** LASSO coefficient profiles of the 16 clinical features. A coefficient profile plot was produced against the log (λ) sequence

Sixty-three variables underwent preliminary univariable screening and 18 variables were found to be statistically significant in the training group were based on independent sample t test, Mann–Whitney–Wilcoxon test, chi-square test or Fisher’s exact test at the appropriate time with *P* < 0.05 for EPL vs. OP, including age, BMI, IgM, C4, TPO-Ab, ACA, β2GP1, LA, HCY, 2 h-BG, 2 h-INS, HDL, CHR, LHR, PNR, induced abortion, TPs, and PLs (Table 2). Then, the 18 variables move to the LASSO logistic regression model in the training set which found 16 variables (age, BMI, IgM, C4, TPO-Ab, ACA, β2GP1, LA, HCY, 2 h-BG, HDL, CHR, PNR, induced abortion, TPs, and PLs) to be predictable (Fig. 2A and B).

Subsequently, the 18 variables selected by univariate screening move to multivariate logistic regression which found that 9 EPL–related features [Age (OR: 1.07; 95% CI: 1.01 to 1.15), BMI (OR: 1.17; 95% CI: 1.00 to 1.37), PLs (OR: 3.56; 95% CI: 1.31 to 9.69), IgM (OR: 1.92; 95% CI: 1.13 to 3.26), HCY (OR: 1.20; 95% CI: 1.06 to 1.36), LHR (OR: 1.47; 95% CI: 1.03 to 2.11), PNR (OR: 1.02; 95% CI: 1.01 to 1.04), induced abortion (OR: 6.64; 95% CI: 1.75 to 25.23), and ACA (OR: 5.11; 95% CI: 1.40 to 18.67)] were identified as independent predictors of subsequent EPL in women with prior RPL (Table 3).

**Table 3:** A multivariate logistic regression found the risk factors for subsequent EPL in women with RPL

Exposure	Univariable OR (95%CI) *P*-value	Multivariable OR (95%CI) *P*-value
Age (year)	1.08 (1.02, 1.14) 0.0134	**1.07 (1.01, 1.15) 0.0387**
BMI (kg/m2)	1.08 (1.00, 1.17) 0.0439	**1.17 (1.00, 1.37) 0.0463**
Total pregnancy numbers	1.24 (1.02, 1.52) 0.0343	1.52 (0.24, 1.13) 0.0999
Pregnancy loss numbers	1.53 (1.13, 2.08) 0.0059	**3.56 (1.31, 9.69) 0.0131**
IG-M(g/L)	1.42 (1.01, 1.99) 0.0421	**1.92 (1.13, 3.26) 0.0157**
C4 (g/L)	2.27 (1.80, 6.50) 0.0249	2.38 (0.45, 12.48) 0.3048
HCY (umol/L)	1.21 (1.12, 1.30) < 0.0001	**1.20 (1.06, 1.36) 0.0042**
2 h-BG (mmol/L)	1.20 (1.01, 1.42) 0.0371	0.95 (0.69, 1.31) 0.7508
2 h-INS (mmol/L)	1.01 (1.00, 1.01) 0.0696	1.00 (0.99, 1.01) 0.6232
HDL (mmol/L)	0.21 (0.10, 0.45) < 0.0001	2.15 (0.37, 12.38) 0.3912
CHR	1.57 (1.17, 2.10) 0.0023	1.10 (0.53, 2.28) 0.7985
LHR	1.58 (1.11, 2.24) 0.0102	**1.47 (1.03, 2.11) 0.0353**
PNR	1.01 (1.00, 1.02) 0.0326	**1.02 (1.01, 1.04) 0.0091**
Induced abortion		
No	1.0	1.0
Yes	2.48 (1.39, 4.42) 0.0021	**6.64 (1.75, 25.23) 0.0055**
TPO-Ab		
Negative	1.0	1.0
Positive	2.04 (1.15, 3.62) 0.0155	1.38 (0.45, 4.27) 0.5764
ACA		
Negative	1.0	1.0
Positive	5.30 (2.55, 11.00) < 0.0001	**5.11 (1.40, 18.67) 0.0135**
β2GP1		
Negative	1.0	1.0
Positive	3.27 (1.37, 7.80) 0.0078	2.40 (0.54, 10.73) 0.2501
LA		
Negative	1.0	1.0
Positive	4.74 (1.93, 11.65) 0.0007	1.11 (0.21, 5.86) 0.8978

*Abbreviations*: *BMI *Body mass index, *IgM* Immunoglobulin M, *C4* Complement C4, *HCY* Homocysteine, *2 h-BG* 2-hour postprandial blood glucose, *2 h-INS* 2-hour postprandial insulin, *HDL* High-density lipoprotein, *CHR* Cholesterol to high-density lipoprotein ratio, *LHR* Low-density lipoprotein to high-density lipoprotein ratio, *PNR* Platelet to neutrophilic ratio, *TPO-Ab* Thyroid peroxidase antibodies, *ACA* Anti cardiolipin antibody, *β2GP1* β2-glycoprotein 1, *LA* Lupus anticoagulant, *OR* Odds ratios, *CI* Confidence interval

### Construction and validation of prediction models

Subsequent EPL prediction model was constructed in the training group using GLM, GBM, RF, and DP with 16 variables selected by LASSO logistic. The four models’ performance results including AUC, AUCPR, logloss, mean per-class error, RMSE and MSE are shown in Table 4. Overall, the GBM model achieved the best performance, with the highest AUC (0.805) and AUCPR (0.783).

**Table 4:** Performance results of four models in the training group based on 16 variables

Training group	GLM	GBM	RF	DP
Mean square error (MSE)	0.1391	0.0701	0.1592	0.0644
Root mean square error (RMSE)	0.3729	0.2648	0.3990	0.2537
LogLoss	0.4406	0.2587	0.9832	0.2124
Mean Per-Class Error	0.2814	0.0728	0.3300	0.0911
**AUC (Area Under the ROC Curve)**	**0.7760**	**0.8053**	**0.7046**	**0.7775**
AUCPR (Area Under the Precision-Recall Curve)	0.6019	0.7831	0.5141	0.7502

*GLM* Generalize linear model, *GBM* Gradient boosting machine, *RF* Random forest, *DP* Deep learning

**Fig. 3:**
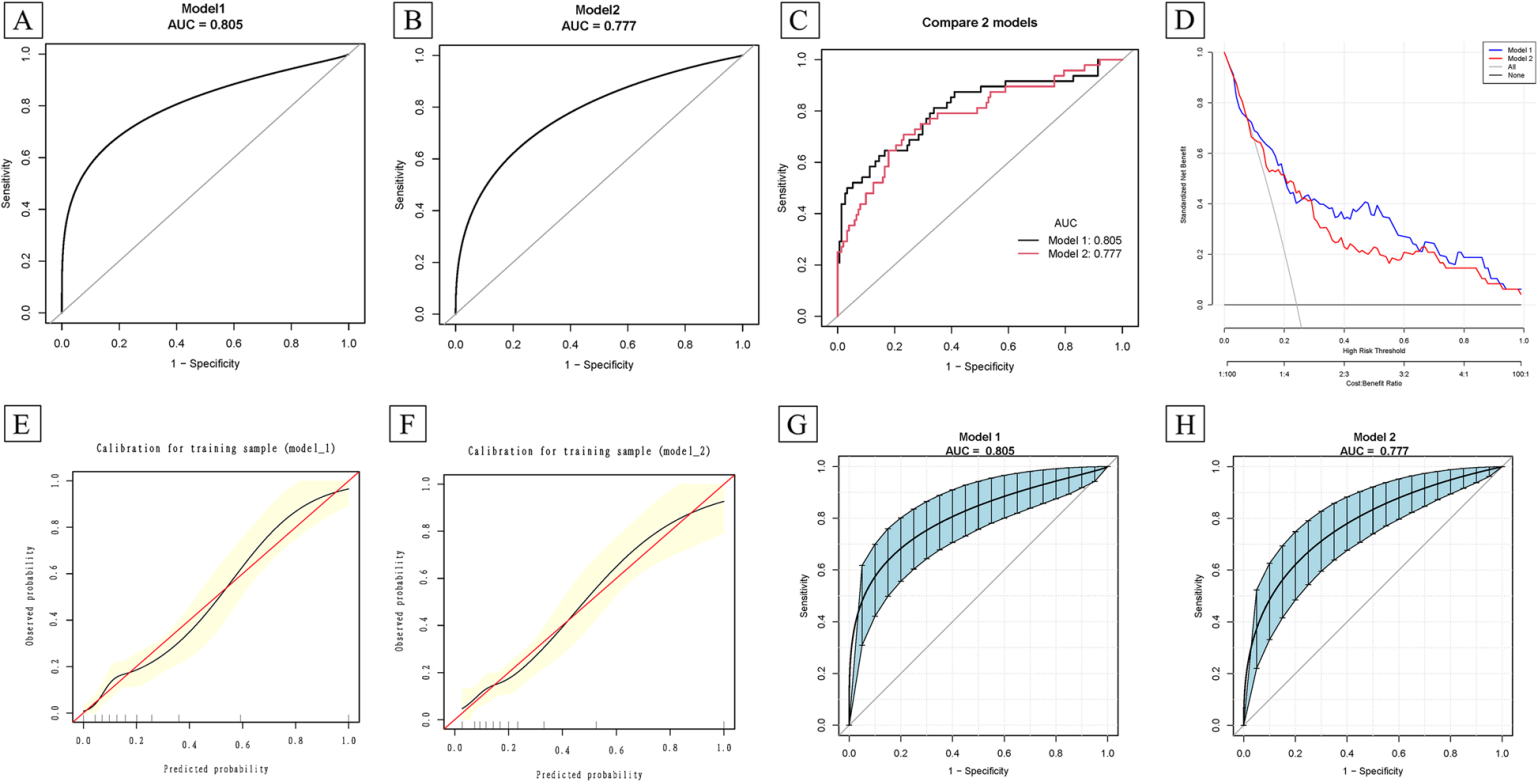
The ROC, DCA, calibration plots, and 95% confidence intervals in the training group for the 16-variables and 9-variables model using GBM. Model 1: 16 variables prediction model; Model 2: 9 variables prediction model. **A-B** ROC curves for models 1 and 2. **C** Comparison of ROC curves between model 1 and model 2. **D **DCA for model 1 and model 2. The y-axis represents the standardized net benefit (sNB), the X-axis represents the threshold probability. The cost-benefit ratio is also shown below the DCA. The black line represents the net benefit when all subjects not occured EPL, and the gray line is the net benefit at each risk threshold when all subjects occurred EPL. The blue and red lines are the net benefits of the risk probabilities estimated by models 1 and 2 at the risk threshold. **E-F** Calibration curves for model 1 and model 2. **G-H** The 95% confidence intervals for model 1 and model 2

Then we used the 9 variables screened again by multivariate regression to construct GBM prediction models in the training group, and compared the prediction performance using 16 and 9 variables, respectively. The results showed that the use of 9 variables did not significantly reduce the prediction performance in the training group, the AUC in 16-variable and 9-variable models were 0.805 (95%CI 0.716, 0.878) and 0.777 (95%CI 0.690, 0.853), *P* > 0.05 (Table 5; Fig. 3A-C). The threshold probability of the DCA is 28% and the corresponding net benefit is 0.44 in 16-variable model, 23% and the corresponding net benefit is 0.48 in 9-variable model. It indicates that two models improve the benefit, and there was no significant difference between the two models (Fig. 3D). Figure 3E-F shows the calibration curve, which suggested that subsequent EPL by 16-variable and 9-variable model were essentially accurate, the Hosmer–Lemeshow test p-value of the two models were 0.607 and 0.559. Figure 3G-H shows the 95% CI in the training group for 16-variable and 9-variable model.

**Table 5:** Comparative analyses in models constructed using 16-variable and 9-variable in training group with GBM

Test	Model 1	Model 2	*P*(compare)
ROC area (AUC)	0.805	0.777	0.1498
95%CI low	0.7159	0.6895	
95%CI upp	0.8777	0.8531	
Specificity	0.8344	0.7682	
Sensitivity	0.6458	0.7083	
Accuracy	0.7889	0.7538	
Positive-LR	3.9008	3.0560	
Negative-LR	0.4244	0.3797	
Diagnose-OR	9.1906	8.0490	
N-for-diagnose	2.0822	2.0984	
Postive-pv	0.5536	0.4928	
Negative-pv	0.8811	0.8923	

Model 1: 16-variables: age, BMI, induced abortion, TPs, PLs, IgM, C4, TPO-Ab, ACA, β2GP1, LA, HCY, 2 h-BG, HDL, CHR, and PNR

Model 2: 9-variables: age, BMI, induced abortion, PLs, IgM, ACA, HCY, LHR, and PNR

**Fig. 4:**
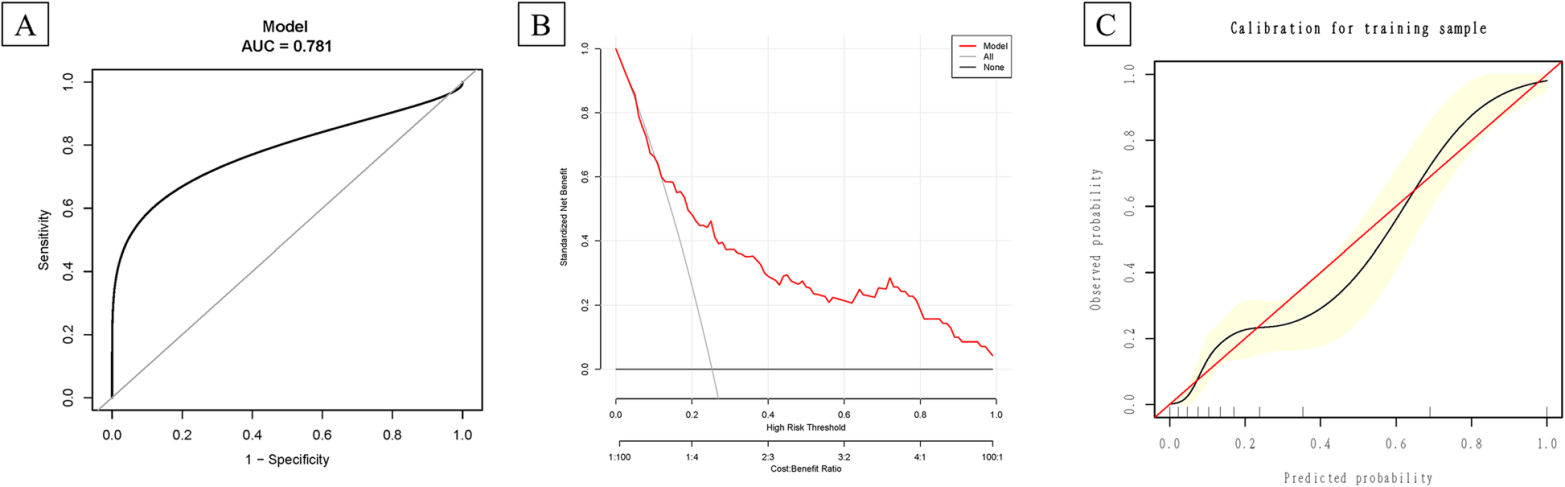
The ROC, DCA, and calibration plots in the validation group for the 9-variables using GBM. **A** ROC curves for GBM model in the validation group. **B** DCA for GBM model in the validation group. The y-axis represents the standardized net benefit (sNB), the X-axis represents the threshold probability. The cost-benefit ratio is also shown below the DCA. The black line represents the net benefit when all subjects not occurred EPL, and the gray line is the net benefit at each risk threshold when all subjects occurred EPL. The red line is the net benefits of the risk probabilities estimated at the risk threshold. **C** Calibration curves for GBM model in the validation group

Because the 16-variable model did not significantly improve predictive power over the 9-variable model, we used the 9-variable model for internal validation based on the validation group. The predictive model displayed a well discrimination performance in the validation group, with an AUC value of 0.781 (95%CI 0.702, 0.843) (Fig. 4A). The DCA showed the model performed well and was feasible for making beneficial clinical decisions (Fig. 4B). Calibration curves revealed the goodness of fit between the predicted values by the model and the actual values, the Hosmer–Lemeshow test was 7.427, and *P* = 0.505 (Fig. 4C).

**Fig. 5:**
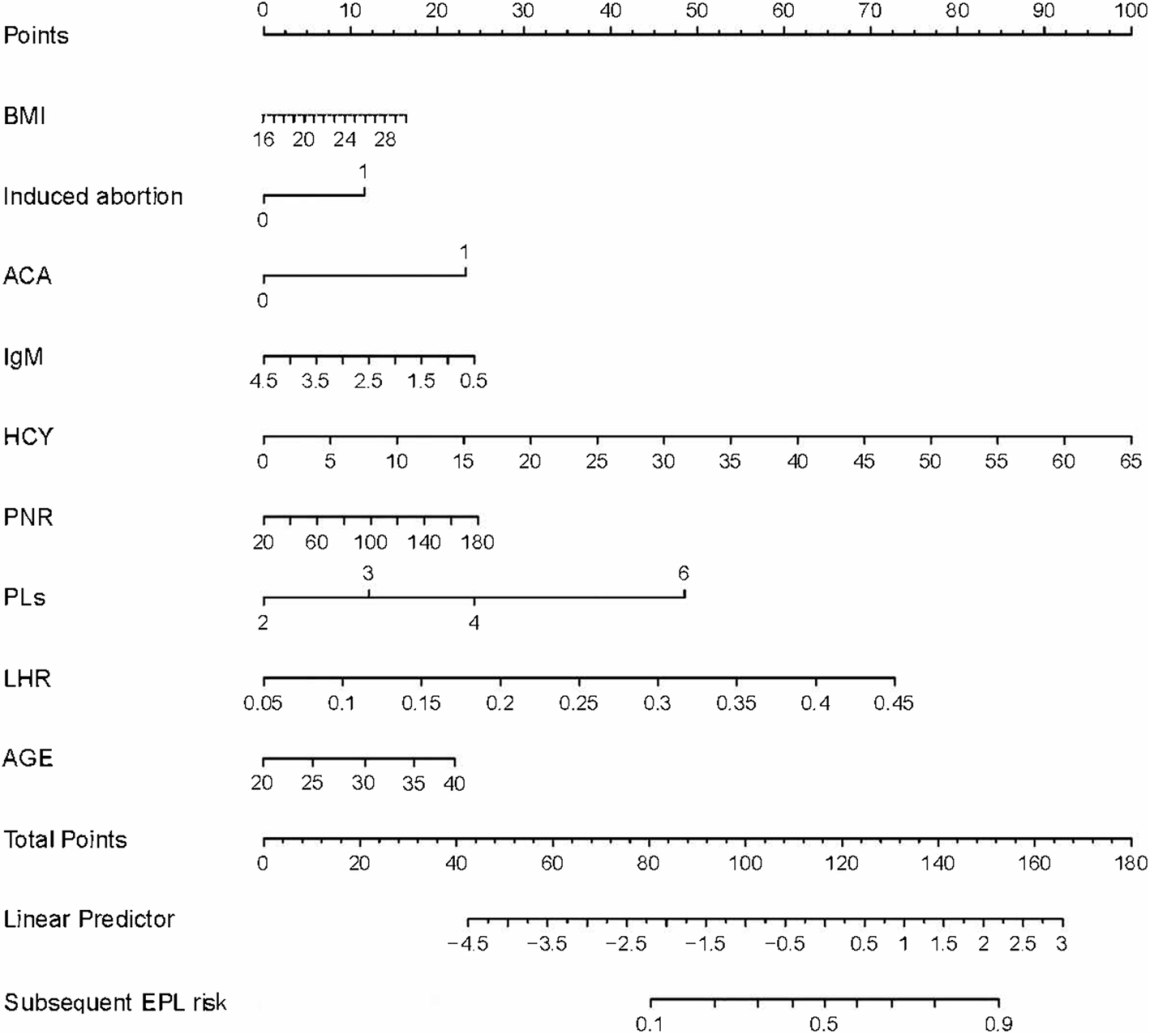
Nomogram for the subsequent EPL prediction of RPL with 9 variables, which including age, BMI, PLs, induced abortion, ACA, HCY, IgM, LHR, and PNR

Finally, 9 variables were finally selected for nomogram presentation in Fig. 5. A total score was obtained by adding matching points for each parameter in the nomogram to evaluate subsequent EPL possibility. The prediction model calculation formula was as follows: subsequent EPL prediction model score = -10.0695 + 0.03738*age + 0.12240*BMI + 0.90452*induced abortion + 1.05194*ACA + 0.62670*IgM + 0.86737*LHR + 0.15993*HCY + 0.02297*PNR + 0.51565*PLs.

## Discussion

The factors affecting the pregnancy outcome of RPL patients are complex and diverse, but it is worth mentioning that a comprehensive review of guidelines states that genetic thrombophilia, vaginal infections, and immunologic and male factors of infertility are not recommended as part of routine RPL investigations and there is also some controversy about the need for ovarian reserve testing, thyroid disease, screening for diabetes or hyperhomocysteinemia, measurement of prolactin levels, and endometrial biopsy [20]. In our study, we first compared the accuracy of multiple machine learning algorithms (GLM, GBM, RF and DP) in predicting subsequent recurrent EPL in patients with RPL, using demographic information and multiple clinical parameters of women with RPL before pregnancy. Subsequently, the best prediction model was used to find that there were no detailed differences in the prediction models constructed with 16 variables and 9 variables. Meanwhile, the 9-variable model displayed a well discrimination performance in the validation group, and the DCA showed the model performed well and was feasible for making beneficial clinical decisions. Calibration curves revealed the goodness of fit between the predicted values by the model and the actual values, the Hosmer–Lemeshow test was 7.427, and *P* = 0.505. The 9 variables include age, BMI, PLs, induced abortion, ACA, HCY, IgM, LHR, and PNR. Our study brings forward the risk assessment of subsequent EPL in women with RPL before pregnancy, which has very important clinical implications.

The association between female age and RPL has been consistently demonstrated in several studies. Studies have shown that couples should start trying to conceive when the woman is 31 or less to have at least a 90% chance of having a two-child family, and if IVF is not feasible, couples should start planning no later than 27. In order to achieve a one-child family, couples should start trying before the age of 32, or 35 if IVF is an option [21]. There are also variations in the threshold of BMI for pregnancy. Zhang et al. reported that, a BMI of 24.0 kg/m^2^ or greater was associated with an increased risk of RPL, but Lo and colleagues demonstrated that maternal obesity (BMI ≥ 30.0 kg/m^2^) significantly increased the risk of miscarriage in couples with unexplained RPL and there was no increased risk in women with overweight and underweight [22, 23]. The conflicting results may due to differences in study design, varying definitions of RPL and BMI ranges. A systematic review and meta-analysis found that the maternal BMI of women with a history of RPL is significantly higher than the BMI of controls, mean difference 0.7 kg/m^2^ [95% CI 0.2–1.3]. It is recommended that BMI be discussed as part of preconception and abortion counseling [24].

We found that for patients with RPL, previous induced abortion increased the risk of RPL recurrence, however previous studies have found that the risk of spontaneous abortion decreases with the increase in the number of induced abortions. This is not consistent with our results. The possible reason is the reference population was derived from all female workers in the Jinchang cohort in China, most of whom had normal reproductive function [25]. In addition, recent studies have found that for IVF patients, termination, miscarriage, ectopic pregnancy, or prior live birth does not compromise subsequent live birth and perinatal outcomes [26]. We also found that the risk of RPL recurrence increased with the number of previous miscarriages. Some studies have found that ≥ 4 previous miscarriages increase the cumulative clinical pregnancy loss rate and reduce the cumulative live birth rate in young women [27], however other studies found that the risk of further miscarriage following two or three RPLs is similar [28]. In a nested cohort, it was demonstrated that the number of prior miscarriages was a determinant both for time to live birth and cumulative incidence of live birth [29, 30]. It is worth noting that for secondary unexplained RPL, only consecutive pregnancy losses after the birth influenced the subsequent prognosis, while the number of losses prior to the birth did not affect the prognosis in the next pregnancy [31].

The negative effects of elevated HCY levels on pregnancy are well known, which is associated with a variety of pregnancy complications, such as preeclampsia (PE), early PL (EPL), placental abruption (PA), intrauterine growth restriction (IUGR) and venous thrombosis [32]. Approximately one third of spontaneous abortion before 20 weeks’ gestation are associated with elevated HCY levels [33]. A longitudinal study based on Chinese population has explored the reference intervals of HCY in three periods of pregnancy, which provides a basis for the management and detection of HCY in Chinese women during pregnancy [34]. However, most studies on the relationship between HCY and pregnancy diseases have focused on the first trimester, ignoring the effect of HCY before pregnancy. Research from our team found that for women with a previous miscarriage, HCY can increase the uterine artery resistance in the non-pregnant state and is associated with the abortion rate of subsequent pregnancy [35]. The present study found that pre-pregnancy HCY plays an important role in the recurrence of RPL in women based on pre-pregnancy data, suggesting that pre-detection of HCY levels in women trying to become pregnant has a positive effect on preventing the recurrence of RPL.

Antiphospholipid antibodies (aPLs) are the leading causes of adverse pregnancy outcomes (APOs). A cluster analysis found that patients with triple antibodies or high-risk lupus characteristics were prone to occurred gestational hypertension and premature delivery. Isolated LA or ACA/aβ2GPI positivity were found to be more frequently associated with early-stage fetal loss [36]. Takeshita et al. found that the only risk factor for persistently positive ACA antibodies is a high antibody titer during the initial test. When the ACA antibody titer in the initial test exceeds the cut-off value (ACA - IgG antibodies > 15 U/mL and ACA - IgM antibodies > 11 U/mL), treatment can be initiated immediately [37]. The complement system has attracted attention as a potential mediator of pathogenic mechanisms induced by aPL. Complement C3 and C4 serum levels were assessed in several cohorts of pregnant patients with APS and/or aPL, these studies have yielded inconsistent results, while some studies have come to find a correlatio, other studies have not revealed a prognostic role for the complement in relation to pregnancy morbidity among aPL-positive women [38–40]. Our study reconfirmed the important effect of positive aPLs and C4 on the outcome of the next pregnancy of RPL patient. But a meta-analysis found that the presence of positive aPL neither decreased clinical pregnancy rate and live birth rate, nor increased miscarriage rate in women undergoing IVF, which is differed from the opinion of clinical practice [41].

Glucose and lipid metabolism levels are not included in the routine screening program of RPL patients in the current guidelines. In this study, 2-hour postprandial glucose and 2-hour postprandial insulin were significantly elevated in women with subsequent EPL, however, there was no significant difference in FBG and FINS between the EPL and OP groups. Study have found that 2-h postprandial glycemia level is more precise than fasting glycemia for type 2 diabetes [42]. As early as 10 years ago, researchers had found that the 1-, 2-, and 3-hour plasma glucose and insulin levels were significantly higher in women with RPL (more than 2 PLs) as compared to controls [43]. Numerous studies associate abnormal glucose metabolism in the endometrium with a higher risk of adverse pregnancy outcomes [44]. Furthermore, several studies have linked altered levels of lipids and a higher risk of adverse pregnancy outcomes, which is visible in patients with recurrent miscarriage (RM) [45, 46], HDL concentrations were lower in women with RM and together with the abdominal obesity were the most frequent components of the RM profile [47]. Subsequently, the study by Depciuch et al. reconfirmed that changes in the metabolomic and lipidomic pathways may be potential risk factors as well as therapeutic targets for RM [48]. In addition, a retrospective cohort study found serum lipid levels were associated with treatment outcomes in women undergoing assisted reproduction, higher HDL-C was associated with greater numbers of oocytes retrieved, higher live birth rates, and lower miscarriage rates [49]. Besides, in long-term follow-up, the researchers found that females with history of PL were experienced more prediabetes (50% vs. 45.5%), diabetes (28.9% vs. 21.3%), and metabolic syndrome (70% vs. 60.1%) than females without such history [50]. This also revealed the interaction between metabolism and RPL.

The role of systemic inflammatory reactions in the pathogenesis of EPL has been confirmed in several studies [51–54]. Inflammatory markers from complete blood count (CBC), such as platelet lymphocyte ratio (PLR), neutrophil-lymphocyte ratio (NLR), neutrophil to monocyte ratio (NMR), lymphocyte to monocyte ratio (LMR) and platelet to neutrophil ratio (PNR) are readily available [55]. This study we found that elevated PNR was a risk factor for EPL in RPL patients in their next pregnancy, and no statistically significant differences were found in the remaining inflammatory markers between the EPL and OP groups. However, the levels of PNR in different diseases are found to be inconsistent. In reproductive events, lower PNR were associated with early natural menopause [56]. For mothers with hypertensive disorders of pregnancy, neonatal Apgar scores at 1 and 5 min of birth were positively associated with PNR [57]. For patients with ST-elevation myocardial infarction, a nonlinear relationship was found between the PNR and major adverse cardiovascular events, which was positively associated with the PNR when the PNR exceeded threshold [58]. For patients with ovarian cancer, PNR were independent prognostic indicators of poor relapse-free survival [59].

However, our study still has some limitations. First, there are some missing data and it is necessary to evaluate the characteristics of RPL women more comprehensively, such as blood pressure, waist circumference and hip circumference, and register more detailed reproductive history and examination results. Second, the inflammatory and immune status of the endometrium are thought to be closely related to RPL, but such data were lacking in this study. Finally, this study is a single-center study and lacks external validation, the model presented here needs further study with more multi-center clinical data. Based on the above shortcomings, our team is carrying out a cohort study of RPL patients to observe and record the whole process of RPL patients from the first visit to the subsequent pregnancy outcome in detail, to provide more accurate clinical treatment for RPL patients.

## Conclusions

Our study innovated the use of pre-pregnancy demographic data and clinical laboratory indicators to predict subsequent EPL in RPL patients, which has important clinical implications. Age, BMI, PLs, induced abortion, ACA, HCY, IgM, LHR, and PNR are the key factors affecting subsequent EPL.

### Supplementary Information

Supplementary Material 1.

Supplementary Material 2.

## Data Availability

No datasets were generated or analysed during the current study.
