# Supplementary material for: Predicting risk of the subsequent early pregnancy loss in women with recurrent pregnancy loss based on preconception data
Source: BMC Womens Health. 2024 Jul 2;24:381. doi: 10.1186/s12905-024-03206-9 (PMC11218098; doi:10.1186/s12905-024-03206-9)
Supplement: Supplementary file 1 — Supplementary Material 1. [file 12905_2024_3206_MOESM1_ESM.docx]

Supplementary Table 1. The details of statistical methods for each variable between early pregnancy loss and ongoing pregnancy group, the number and percentage of missing data.

| Variables | Statistical method | Missing data number | Missing data percentage |
| --- | --- | --- | --- |
| Age (year) | T test | 0 | 0.00% |
| BMI (kg/m^2^) | T test | 12 | 1.98% |
| Total pregnancy numbers | T test | 0 | 0.00% |
| Pregnancy loss numbers | T test | 0 | 0.00% |
| Education | chi-square test | 1 | 0.01% |
| Primary school (n, %) |  |  |  |
| Secondary school (n, %) |  |  |  |
| Bachelor degree (n, %) |  |  |  |
| Graduate degree (n, %) |  |  |  |
| Ethnic | chi-square test | 5 | 0.83% |
| Han nationality (n, %) |  |  |  |
| Others (n, %) |  |  |  |
| Menarche (year) | T test | 33 | 5.45% |
| Menstrual cycle | chi-square test | 0 | 0.00% |
| regular (n, %) |  |  |  |
| irregular (n, %) |  |  |  |
| Pelvic surgery | chi-square test | 0 | 0.00% |
| No (n, %) |  |  |  |
| Yes (n, %) |  |  |  |
| Preconception treatments | chi-square test | 0 | 0.00% |
| No (n, %) |  |  |  |
| Yes (n, %) |  |  |  |
| Induced abortion | chi-square test | 0 | 0.00% |
| No (n, %) |  |  |  |
| Yes (n, %) |  |  |  |
| Live birth | chi-square test | 0 | 0.00% |
| No (n, %) |  |  |  |
| Yes (n, %) |  |  |  |
| Pregnancy type | chi-square test | 0 | 0.00% |
| Primary (n, %) |  |  |  |
| Secondary (n, %) |  |  |  |
| TSH (uIU /mL) | T test | 37 | 6.11% |
| TG-Ab | chi-square test | 40 | 6.61% |
| Negative (n, %) |  |  |  |
| Positive (n, %) |  |  |  |
| TPO-Ab | chi-square test | 40 | 6.61% |
| Negative (n, %) |  |  |  |
| Positive (n, %) |  |  |  |
| IG-G (g/L) | T test | 0 | 0.00% |
| IG-A (g/L) | T test | 0 | 0.00% |
| IG-M(g/L) | T test | 0 | 0.00% |
| C3 (g/L) | T test | 7 | 1.15% |
| C4 (g/L) | Mann–Whitney–Wilcoxon test | 7 | 1.15% |
| ANA | chi-square test | 39 | 6.44% |
| Negative (n, %) |  |  |  |
| Positive (n, %) |  |  |  |
| ACA | chi-square test | 32 | 5.29% |
| Negative (n, %) |  |  |  |
| Positive (n, %) |  |  |  |
| β2GP1 | chi-square test | 32 | 5.29% |
| Negative (n, %) |  |  |  |
| Positive (n, %) |  |  |  |
| LA | chi-square test | 32 | 5.29% |
| Negative (n, %) |  |  |  |
| Positive (n, %) |  |  |  |
| D-dimer (mg/L) | Mann–Whitney–Wilcoxon test | 0 | 0.00% |
| HCY (umol/L) | Mann–Whitney–Wilcoxon test | 18 | 2.98% |
| 25(OH)D (ng/ml) | T test | 22 | 3.63% |
| FBG (mmol/L) | T test | 13 | 2.14% |
| FINS (mU/L) | Mann–Whitney–Wilcoxon test | 13 | 2.14% |
| HOMA-IR | T test | 13 | 2.14% |
| FCP (ng/ml) | T test | 19 | 2.14% |
| 2h-BG (mmol/L) | T test | 18 | 2.98% |
| 2h-INS (mU/L) | Mann–Whitney–Wilcoxon test | 18 | 2.98% |
| 2h-CP (ng/ml) | T test | 18 | 2.98% |
| CHO (mmol/L) | T test | 0 | 0.00% |
| TG (mmol/L) | Mann–Whitney–Wilcoxon test | 0 | 0.00% |
| HDL (mmol/L) | T test | 0 | 0.00% |
| LDL (mmol/L) | T test | 0 | 0.00% |
| CHR | T test | 0 | 0.00% |
| THR | Mann–Whitney–Wilcoxon test | 0 | 0.00% |
| LHR | T test | 0 | 0.00% |
| WBC (×109) | T test | 0 | 0.00% |
| NE# (×109) | T test | 0 | 0.00% |
| LY# (×109) | T test | 0 | 0.00% |
| MO# (×109) | T test | 0 | 0.00% |
| RBC (×1012) | T test | 0 | 0.00% |
| HGB (g/L) | T test | 0 | 0.00% |
| PLT (×109) | T test | 0 | 0.00% |
| LWR | T test | 0 | 0.00% |
| NLR | T test | 0 | 0.00% |
| NMR | T test | 0 | 0.00% |
| LMR | T test | 0 | 0.00% |
| PWR | T test | 0 | 0.00% |
| PNR | T test | 0 | 0.00% |
| PLR | T test | 0 | 0.00% |
| PMR | T test | 0 | 0.00% |
| ALT (U/L) | T test | 0 | 0.00% |
| AST (U/L) | T test | 0 | 0.00% |
| AST/ALT | T test | 0 | 0.00% |
| SUR (mmol/L) | T test | 28 | 4.63% |
| SCR (μmol/L) | T test | 28 | 4.63% |
| SUA (μmol/L) | T test | 28 | 4.63% |

Abbreviations: BMI: Body mass index TSH: Thyroid stimulating hormone; TG-Ab: Thyroglobulin antibody; TPO-Ab: Thyroid peroxidase antibodies; ANA: Antinuclear antibody; ACA: Anti cardiolipin antibody; β2GP1: β2-glycoprotein 1; LA: Lupus anticoagulant; IgG: Immunoglobulin G; IgA: Immunoglobulin A; IgM: Immunoglobulin M; C3: Complement C3; C4: Complement C4; HCY: Homocysteine; 25(OH)D: 25-hydroxy-vitamin; FBG (mmol/L); FBG: Fasting blood glucose; FINS: Fasting insulin; HOMA-IR: Homeostasis model assessment of insulin resistance; FCP: Fasting C-peptide; 2h-BG: 2-hour postprandial blood glucose; 2h-INS: 2-hour postprandial insulin; 2h-CP: 2-hour postprandial C-peptide; CHO: Cholesterol; TG: Triglyceride; HDL: High-density lipoprotein; LDL: Low-density lipoprotein; CHR: Cholesterol to high-density lipoprotein ratio; THR: Triglyceride to high-density lipoprotein ratio; LHR: Low-density lipoprotein to high-density lipoprotein ratio; WBC: White blood cell; NE: Neutrophilic; LY: Lymphocyte; MO: Monocytes; RBC: Red blood cell; HGB: Hemoglobin; PLT: Platelet; LWR: Lymphocyte to white blood cell ratio; NLR: Neutrophilic to lymphocyte ratio; NMR: Neutrophilic to monocytes ratio; LMR: Lymphocyte to monocytes ratio; PWR: Platelet to white blood cell ratio; PNR: Platelet to neutrophilic ratio; PLR: Platelet to lymphocyte; PMR: Platelet to monocytes ratio; ALT: Alanine Aminotransferase; AST: Aspartate Transaminase; AST/ALT: Aspartate Transaminase to alanine Aminotransferase; SUR: Serum urea; SCR: Serum creatinine; SUA: Serum uric acid.
