# Supplementary material for: Predicting risk of the subsequent early pregnancy loss in women with recurrent pregnancy loss based on preconception data
Source: BMC Womens Health. 2024 Jul 2;24:381. doi: 10.1186/s12905-024-03206-9 (PMC11218098; doi:10.1186/s12905-024-03206-9)
Supplement: Supplementary file 2 — Supplementary Material 2. [file 12905_2024_3206_MOESM2_ESM.docx]

| Supplementary Table 2. Multiple imputation of missing values. | | | | | | | |
| --- | --- | --- | --- | --- | --- | --- | --- |
| Variables | Original data | Imputation 1 | Imputation 2 | Imputation 3 | Imputation 4 | Imputation 5 | P-value |
| Age (year) | 30.02 ± 3.81 | 30.02 ± 3.81 | 30.02 ± 3.81 | 30.02 ± 3.81 | 30.02 ± 3.81 | 30.02 ± 3.81 | 1.000 |
| BMI (kg/m^2^) | 22.12 ± 2.70 | 22.06 ± 2.79 | 22.05 ± 2.79 | 22.09 ± 2.79 | 22.05 ± 2.79 | 22.06 ± 2.80 | 0.999 |
| Total pregnancy numbers | 2.78 ± 0.97 | 2.78 ± 0.97 | 2.78 ± 0.97 | 2.78 ± 0.97 | 2.78 ± 0.97 | 2.78 ± 0.97 | 1.000 |
| Pregnancy loss numbers | 2.38 ± 0.64 | 2.38 ± 0.64 | 2.38 ± 0.64 | 2.38 ± 0.64 | 2.38 ± 0.64 | 2.38 ± 0.64 | 1.000 |
| Education |  |  |  |  |  |  | 1.000 |
| Primary school (n, %) | 20 (3.47%) | 21 (3.47%) | 19 (3.14%) | 20 (3.31%) | 19 (3.14%) | 22 (3.64%) |  |
| Secondary school (n, %) | 167 (27.60%) | 167 (27.60%) | 171 (28.26%) | 169 (27.93%) | 165 (27.27%) | 168 (27.77%) |  |
| Bachelor degree (n, %) | 389 (64.30%) | 389 (64.30%) | 388 (64.13%) | 388 (64.13%) | 394 (65.12%) | 385 (63.64%) |  |
| Graduate degree (n, %) | 28 (4.63%) | 28 (4.63%) | 27 (4.46%) | 28 (4.63%) | 27 (4.46%) | 30 (4.96%) |  |
| Ethnic |  |  |  |  |  |  | 1.000 |
| Han nationality (n, %) | 546 (91.00%) | 550 (90.91%) | 551 (91.07%) | 551 (91.07%) | 550 (90.91%) | 551 (91.07%) |  |
| Others (n, %) | 54 (9.00%) | 55 (9.09%) | 54 (8.93%) | 54 (8.93%) | 55 (9.09%) | 54 (8.93%) |  |
| Menarche (year) | 13.49 ± 1.20 | 13.53 ± 1.24 | 13.51 ± 1.28 | 13.49 ± 1.27 | 13.50 ± 1.27 | 13.51 ± 1.29 | 0.996 |
| Menstrual cycle |  |  |  |  |  |  | 1.000 |
| regular (n, %) | 527 (87.11%) | 527 (87.11%) | 527 (87.11%) | 527 (87.11%) | 527 (87.11%) | 527 (87.11%) |  |
| irregular (n, %) | 78 (12.89%) | 78 (12.89%) | 78 (12.89%) | 78 (12.89%) | 78 (12.89%) | 78 (12.89%) |  |
| Pelvic surgery |  |  |  |  |  |  | 1.000 |
| No (n, %) | 494 (81.65%) | 494 (81.65%) | 494 (81.65%) | 494 (81.65%) | 494 (81.65%) | 494 (81.65%) |  |
| Yes (n, %) | 111 (18.35%) | 111 (18.35%) | 111 (18.35%) | 111 (18.35%) | 111 (18.35%) | 111 (18.35%) |  |
| Preconception treatments |  |  |  |  |  |  | 1.000 |
| No (n, %) | 190 (31.40%) | 190 (31.40%) | 190 (31.40%) | 190 (31.40%) | 190 (31.40%) | 190 (31.40%) |  |
| Yes (n, %) | 415 (68.60%) | 415 (68.60%) | 415 (68.60%) | 415 (68.60%) | 415 (68.60%) | 415 (68.60%) |  |
| Induced abortion |  |  |  |  |  |  | 1.000 |
| No (n, %) | 532 (87.93%) | 532 (87.93%) | 532 (87.93%) | 532 (87.93%) | 532 (87.93%) | 532 (87.93%) |  |
| Yes (n, %) | 73 (12.07%) | 73 (12.07%) | 73 (12.07%) | 73 (12.07%) | 73 (12.07%) | 73 (12.07%) |  |
| Live birth |  |  |  |  |  |  | 1.000 |
| No (n, %) | 484 (80.00%) | 484 (80.00%) | 484 (80.00%) | 484 (80.00%) | 484 (80.00%) | 484 (80.00%) |  |
| Yes (n, %) | 121 (20.00%) | 121 (20.00%) | 121 (20.00%) | 121 (20.00%) | 121 (20.00%) | 121 (20.00%) |  |
| Pregnancy type |  |  |  |  |  |  | 1.000 |
| Primary (n, %) | 474 (78.35%) | 474 (78.35%) | 474 (78.35%) | 474 (78.35%) | 474 (78.35%) | 474 (78.35%) |  |
| Secondary (n, %) | 131 (21.65%) | 131 (21.65%) | 131 (21.65%) | 131 (21.65%) | 131 (21.65%) | 131 (21.65%) |  |
| TSH (uIU /mL) | 2.59 ± 1.40 | 2.60 ± 1.46 | 2.62 ± 1.49 | 2.59 ± 1.50 | 2.62 ± 1.52 | 2.61 ± 1.50 | 0.997 |
| TG-Ab |  |  |  |  |  |  | 0.721 |
| Negative (n, %) | 481((85.13%) | 510 (84.30%) | 510 (84.30%) | 511 (84.46%) | 514 (84.96%) | 513 (84.79%) |  |
| Positive (n, %) | 84 (14.87%) | 95 (15.70%) | 95 (15.70%) | 94 (15.54%) | 91 (15.04%) | 92 (15.21%) |  |
| TPO-Ab |  |  |  |  |  |  | 0.603 |
| Negative (n, %) | 478 (84.60%) | 509 (84.13%) | 509 (84.13%) | 494 (81.65%) | 499 (82.48%) | 497 (82.15%) |  |
| Positive (n, %) | 87 (15.40%) | 96 (15.87%) | 96 (15.87%) | 111 (18.35%) | 106 (17.52%) | 108 (17.85%) |  |
| IG-G (g/L) | 12.94 ± 2.81 | 12.94 ± 2.81 | 12.94 ± 2.81 | 12.94 ± 2.81 | 12.94 ± 2.81 | 12.94 ± 2.81 | 1.000 |
| IG-A (g/L) | 2.18 ± 0.73 | 2.18 ± 0.73 | 2.18 ± 0.73 | 2.18 ± 0.73 | 2.18 ± 0.73 | 2.18 ± 0.73 | 1.000 |
| IG-M(g/L) | 2.03 ± 0.74 | 2.03 ± 0.74 | 2.03 ± 0.74 | 2.03 ± 0.74 | 2.03 ± 0.74 | 2.03 ± 0.74 | 1.000 |
| C3 (g/L) | 1.14 ± 0.20 | 1.15 ± 0.20 | 1.15 ± 0.21 | 1.15 ± 0.21 | 1.15 ± 0.21 | 1.15 ± 0.21 | 0.969 |
| C4 (g/L) | 0.26 (0.21-0.32) | 0.28 ± 0.28 | 0.29 ± 0.28 | 0.29 ± 0.28 | 0.29 ± 0.28 | 0.30 ± 0.28 | 0.879 |
| ANA |  |  |  |  |  |  | 0.960 |
| Negative (n, %) | 498 (87.99%) | 530 (87.60%) | 532 (87.93%) | 536 (88.60%) | 537 (88.76%) | 540 (89.26%) |  |
| Positive (n, %) | 68 (12.01%) | 75 (12.40%) | 73 (12.07%) | 69 (11.40%) | 68 (11.24%) | 65 (10.74%) |  |
| ACA |  |  |  |  |  |  | 0.976 |
| Negative (n, %) | 512 (89.35%) | 554 (91.57%) | 560 (92.56%) | 557 (92.07%) | 557 (92.07%) | 558 (92.23%) |  |
| Positive (n, %) | 61 (10.65%) | 51 (8.43%) | 45 (7.44%) | 48 (7.93%) | 48 (7.93%) | 47 (7.77%) |  |
| β2GP1 |  |  |  |  |  |  | 0.088 |
| Negative (n, %) | 527 (91.97%) | 563 (93.06%) | 559 (92.40%) | 553 (91.40%) | 550 (90.91%) | 540 (89.26%) |  |
| Positive (n, %) | 46 (8.03%) | 42 (6.94%) | 46 (7.60%) | 52 (8.60%) | 55 (9.09%) | 65 (10.74%) |  |
| LA |  |  |  |  |  |  | 0.795 |
| Negative (n, %) | 528 (92.15%) | 577 (95.37%) | 568 (93.88%) | 568 (93.88%) | 566 (93.55%) | 572 (94.55%) |  |
| Positive (n, %) | 45 (7.85%) | 28 (4.63%) | 37 (6.12%) | 37 (6.12%) | 39 (6.45%) | 33 (5.45%) |  |
| D-dimer (mg/L) | 0.18 (0.12-0.28) | 0.18 (0.12-0.28) | 0.18 (0.12-0.28) | 0.18 (0.12-0.28) | 0.18 (0.12-0.28) | 0.18 (0.12-0.28) | 1.000 |
| HCY (umol/L) | 11.71 ± 5.97 | 11.71 ± 6.84 | 11.65 ± 6.45 | 11.81 ± 6.33 | 11.88 ± 6.82 | 11.94 ± 6.77 | 0.975 |
| 25(OH)D (ng/ml) | 12.01 ± 4.63 | 12.35 ± 5.06 | 12.41 ± 5.14 | 12.23 ± 5.11 | 12.29 ± 5.05 | 12.23 ± 5.05 | 0.822 |
| FBG (mmol/L) | 4.99 ± 0.44 | 4.97 ± 0.47 | 4.96 ± 0.46 | 4.96 ± 0.47 | 4.97 ± 0.46 | 4.97 ± 0.47 | 0.966 |
| FINS (mU/L) | 10.21 (7.13-12.28) | 10.82 ± 6.73 | 10.81 ± 6.74 | 10.82 ± 6.74 | 10.77 ± 6.70 | 10.75 ± 6.74 | 1.000 |
| HOMA-IR | 2.41 ± 1.58 | 2.43 ± 1.63 | 2.42 ± 1.62 | 2.42 ± 1.63 | 2.42 ± 1.62 | 2.41 ± 1.63 | 1.000 |
| FCP (ng/ml) | 1.36 ± 0.73 | 1.41 ± 0.92 | 1.45 ± 0.98 | 1.44 ± 0.98 | 1.46 ± 1.04 | 1.47 ± 1.07 | 0.355 |
| 2h-BG (mmol/L) | 5.67 ± 1.22 | 5.75 ± 1.26 | 5.78 ± 1.28 | 5.78 ± 1.30 | 5.80 ± 1.27 | 5.85 ± 1.33 | 0.289 |
| 2h-INS (mU/L) | 31.03 (23.67-51.64) | 42.40 ± 33.53 | 42.10 ± 33.31 | 41.61 ± 32.95 | 42.13 ± 32.74 | 41.78 ± 32.97 | 0.997 |
| 2h-CP (ng/ml) | 4.48 ± 1.82 | 4.56 ± 2.13 | 4.57 ± 2.06 | 4.56 ± 2.10 | 4.55 ± 2.04 | 4.58 ± 2.10 | 0.960 |
| CHO (mmol/L) | 3.86 ± 0.67 | 3.86 ± 0.67 | 3.86 ± 0.67 | 3.86 ± 0.67 | 3.86 ± 0.67 | 3.86 ± 0.67 | 1.000 |
| TG (mmol/L) | 0.94 (0.73-1.21) | 0.94 (0.73-1.21) | 0.94 (0.73-1.21) | 0.94 (0.73-1.21) | 0.94 (0.73-1.21) | 0.94 (0.73-1.21) | 1.000 |
| HDL (mmol/L) | 1.41 ± 0.31 | 1.41 ± 0.31 | 1.41 ± 0.31 | 1.41 ± 0.31 | 1.41 ± 0.31 | 1.41 ± 0.31 | 1.000 |
| LDL (mmol/L) | 2.45 ± 0.59 | 2.45 ± 0.59 | 2.45 ± 0.59 | 2.45 ± 0.59 | 2.45 ± 0.59 | 2.45 ± 0.59 | 1.000 |
| CHR | 2.85 ± 0.71 | 2.85 ± 0.71 | 2.85 ± 0.71 | 2.85 ± 0.71 | 2.85 ± 0.71 | 2.85 ± 0.71 | 1.000 |
| THR | 0.63 (0.50-0.98) | 0.63 (0.50-0.98) | 0.63 (0.50-0.98) | 0.63 (0.50-0.98) | 0.63 (0.50-0.98) | 0.63 (0.50-0.98) | 1.000 |
| LHR | 1.82 ± 0.59 | 1.82 ± 0.59 | 1.82 ± 0.59 | 1.82 ± 0.59 | 1.82 ± 0.59 | 1.82 ± 0.59 | 1.000 |
| WBC (×109) | 5.88 ± 2.35 | 5.88 ± 2.35 | 5.88 ± 2.35 | 5.88 ± 2.35 | 5.88 ± 2.35 | 5.88 ± 2.35 | 1.000 |
| NE# (×109) | 3.69 ± 1.31 | 3.69 ± 1.31 | 3.69 ± 1.31 | 3.69 ± 1.31 | 3.69 ± 1.31 | 3.69 ± 1.31 | 1.000 |
| LY# (×109) | 1.71 ± 0.47 | 1.71 ± 0.47 | 1.71 ± 0.47 | 1.71 ± 0.47 | 1.71 ± 0.47 | 1.71 ± 0.47 | 1.000 |
| MO# (×109) | 0.27 ± 0.09 | 0.27 ± 0.09 | 0.27 ± 0.09 | 0.27 ± 0.09 | 0.27 ± 0.09 | 0.27 ± 0.09 | 1.000 |
| RBC (×1012) | 4.60 ± 0.39 | 4.60 ± 0.39 | 4.60 ± 0.39 | 4.60 ± 0.39 | 4.60 ± 0.39 | 4.60 ± 0.39 | 1.000 |
| HGB (g/L) | 140.95 ± 12.66 | 140.95 ± 12.66 | 140.95 ± 12.66 | 140.95 ± 12.66 | 140.95 ± 12.66 | 140.95 ± 12.66 | 1.000 |
| PLT (×109) | 222.59 ± 59.09 | 222.59 ± 59.09 | 222.59 ± 59.09 | 222.59 ± 59.09 | 222.59 ± 59.09 | 222.59 ± 59.09 | 1.000 |
| LWR | 0.30 ± 0.08 | 0.30 ± 0.08 | 0.30 ± 0.08 | 0.30 ± 0.08 | 0.30 ± 0.08 | 0.30 ± 0.08 | 1.000 |
| NLR | 2.29 ± 1.00 | 2.29 ± 1.00 | 2.29 ± 1.00 | 2.29 ± 1.00 | 2.29 ± 1.00 | 2.29 ± 1.00 | 1.000 |
| NMR | 14.24 ± 4.91 | 14.24 ± 4.91 | 14.24 ± 4.91 | 14.24 ± 4.91 | 14.24 ± 4.91 | 14.24 ± 4.91 | 1.000 |
| LMR | 6.63 ± 2.08 | 6.63 ± 2.08 | 6.63 ± 2.08 | 6.63 ± 2.08 | 6.63 ± 2.08 | 6.63 ± 2.08 | 1.000 |
| PWR | 40.12 ± 12.16 | 40.12 ± 12.16 | 40.12 ± 12.16 | 40.12 ± 12.16 | 40.12 ± 12.16 | 40.12 ± 12.16 | 1.000 |
| PNR | 66.38 ± 26.91 | 66.38 ± 26.91 | 66.38 ± 26.91 | 66.38 ± 26.91 | 66.38 ± 26.91 | 66.38 ± 26.91 | 1.000 |
| PLR | 138.07 ± 45.69 | 138.07 ± 45.69 | 138.07 ± 45.69 | 138.07 ± 45.69 | 138.07 ± 45.69 | 138.07 ± 45.69 | 1.000 |
| PMR | 883.63 ± 321.17 | 883.63 ± 321.17 | 883.63 ± 321.17 | 883.63 ± 321.17 | 883.63 ± 321.17 | 883.63 ± 321.17 | 1.000 |
| ALT (U/L) | 17.85 ± 13.54 | 17.85 ± 13.54 | 17.85 ± 13.54 | 17.85 ± 13.54 | 17.85 ± 13.54 | 17.85 ± 13.54 | 1.000 |
| AST (U/L) | 22.76 ± 9.31 | 22.76 ± 9.31 | 22.76 ± 9.31 | 22.76 ± 9.31 | 22.76 ± 9.31 | 22.76 ± 9.31 | 1.000 |
| AST/ALT | 1.62 ± 0.95 | 1.62 ± 0.95 | 1.62 ± 0.95 | 1.62 ± 0.95 | 1.62 ± 0.95 | 1.62 ± 0.95 | 1.000 |
| SUR (mmol/L) | 4.38 ± 1.16 | 4.40 ± 1.20 | 4.45 ± 1.24 | 4.41 ± 1.22 | 4.37 ± 1.21 | 4.33 ± 1.23 | 0.617 |
| SCR (μmol/L) | 54.53 ± 7.72 | 55.31 ± 8.14 | 55.18 ± 7.95 | 55.05 ± 7.74 | 55.40 ± 8.16 | 55.66 ± 8.19 | 0.295 |
| SUA (μmol/L) | 266.55 ± 60.97 | 260.27 ± 68.06 | 260.04 ± 68.24 | 257.54 ± 66.80 | 256.92 ± 66.90 | 257.46 ± 67.68 | 0.182 |

Abbreviations: BMI: Body mass index TSH: Thyroid stimulating hormone; TG-Ab: Thyroglobulin antibody; TPO-Ab: Thyroid peroxidase antibodies; ANA: Antinuclear antibody; ACA: Anti cardiolipin antibody; β2GP1: β2-glycoprotein 1; LA: Lupus anticoagulant; IgG: Immunoglobulin G; IgA: Immunoglobulin A; IgM: Immunoglobulin M; C3: Complement C3; C4: Complement C4; HCY: Homocysteine; 25(OH)D: 25-hydroxy-vitamin; FBG (mmol/L); FBG: Fasting blood glucose; FINS: Fasting insulin; HOMA-IR: Homeostasis model assessment of insulin resistance; FCP: Fasting C-peptide; 2h-BG: 2-hour postprandial blood glucose; 2h-INS: 2-hour postprandial insulin; 2h-CP: 2-hour postprandial C-peptide; CHO: Cholesterol; TG: Triglyceride; HDL: High-density lipoprotein; LDL: Low-density lipoprotein; CHR: Cholesterol to high-density lipoprotein ratio; THR: Triglyceride to high-density lipoprotein ratio; LHR: Low-density lipoprotein to high-density lipoprotein ratio; WBC: White blood cell; NE: Neutrophilic; LY: Lymphocyte; MO: Monocytes; RBC: Red blood cell; HGB: Hemoglobin; PLT: Platelet; LWR: Lymphocyte to white blood cell ratio; NLR: Neutrophilic to lymphocyte ratio; NMR: Neutrophilic to monocytes ratio; LMR: Lymphocyte to monocytes ratio; PWR: Platelet to white blood cell ratio; PNR: Platelet to neutrophilic ratio; PLR: Platelet to lymphocyte; PMR: Platelet to monocytes ratio; ALT: Alanine Aminotransferase; AST: Aspartate Transaminase; AST/ALT: Aspartate Transaminase to alanine Aminotransferase; SUR: Serum urea; SCR: Serum creatinine; SUA: Serum uric acid.
